# Pulmonary Nodule Detection Based on Multiscale Feature Fusion

**DOI:** 10.1155/2022/8903037

**Published:** 2022-12-21

**Authors:** Yue Zhao, Zhongyang Wang, Xinyao Liu, Qi Chen, Chuangang Li, Hongshuo Zhao, Zhiqiong Wang

**Affiliations:** ^1^College of Medicine & Biological Information Engineering, Northeastern University, Shenyang 110169, China; ^2^School of Computer Science & Engineering, Northeastern University, Shenyang 110169, China; ^3^Key Laboratory of Big Data Management and Analytics, Liaoning Province, Northeastern University, Shenyang 110169, China

## Abstract

As cancer with the highest morbidity and mortality in the world, lung cancer is characterized by pulmonary nodules in the early stage. The detection of pulmonary nodules is an important method for the early detection of lung cancer, which can greatly improve the survival rate of lung cancer patients. However, the accuracy of conventional detection methods for lung nodules is low. With the development of medical imaging technology, deep learning plays an increasingly important role in medical image detection, and pulmonary nodules can be accurately detected by CT images. Based on the above, a pulmonary nodule detection method based on deep learning is proposed. In the candidate nodule detection stage, the multiscale features and Faster R-CNN, a general-purpose detection framework based on deep learning, were combined together to improve the detection of small-sized lung nodules. In the false-positive nodule filtration stage, a 3D convolutional neural network based on multiscale fusion is designed to reduce false-positive nodules. The experiment results show that the candidate nodule detection model based on Faster R-CNN integrating multiscale features has achieved a sensitivity of 98.6%, 10% higher than that of the other single-scale model, the proposed method achieved a sensitivity of 90.5% at the level of 4 false-positive nodules per scan, and the CPM score reached 0.829. The results are higher than methods in other works of literature. It can be seen that the detection method of pulmonary nodules based on multiscale fusion has a higher detection rate for small nodules and improves the classification performance of true and false-positive pulmonary nodules. This will help doctors when making a lung cancer diagnosis.

## 1. Introduction

Lung cancer is cancer with high mortality in the world. Compared with other cancers, lung cancer has the characteristics of shorter onset time, higher malignancy, and difficult to diagnose accurately in the early stage. The early clinical manifestation of lung cancer is pulmonary nodules, and the analysis of pulmonary nodules by computer tomography (CT) can obtain the characteristics of nodules comprehensively [1]. Therefore, the detection of pulmonary nodules based on CT images is an important method for the early detection of lung cancer. With the rapid development of CT imaging technology and smart healthcare management system [2], in order to help radiologists detect pulmonary nodules from a large number of CT images [3, 4], many researchers have developed computer-aided detection systems. Abbreviations in the text are shown in Table 1.

Computer-aided diagnosis systems for pulmonary nodule detection and segmentation are usually divided into two stages: candidate pulmonary nodule detection and false-positive nodule filtering. When candidate nodule detection is performed, the whole CT image is usually scanned to obtain a large number of candidate nodules, and these candidate nodules contain a large number of false-positive nodules. Therefore, the results at this stage are not only highly sensitive but also have a high false-positive rate. Next, false-positive nodules were filtered on the candidate nodules obtained in the previous stage, which made the results with high sensitivity and low false-positive rate, and the whole task of pulmonary nodule detection was completed. Traditional computer-aided systems for pulmonary nodule detection have achieved good results, but they are difficult to accurately describe these variable nodules by using low-level features extracted manually [5]. The detection time was too long because of the need to segment the lung tissue first. The traditional segmentation of lung nodules is mainly semiautomatic segmentation, which usually communicates with radiologists to determine the approximate position of lung nodules, and then realizes automatic segmentation through computer technology. Although this method of lung nodule segmentation has high accuracy, it requires human participation and cannot achieve end-to-end operation.

In recent years, deep learning has developed rapidly, especially in the field of image processing. Because of its strong ability of feature self-learning and expression, deep learning has gradually become the focus of research. With the establishment of various image databases, convolutional neural network (CNN) has gradually become a standard feature extractor to complete computer vision tasks in various fields [6] and has also made many excellent results in the field of medical image analysis [7–11]. Faster R-CNN is a general target detection model based on CNN [12], which replaces the traditional method of extracting candidate targets by Selective Search with a region proposal network (RPN), which greatly improves the detection speed and realizes end-to-end in the true sense.

Computer-aided diagnosis of pulmonary nodules using deep learning methods can automatically detect and segment pulmonary nodules, which can help radiologists reduce their work pressure. However, due to the small proportion of pulmonary nodules in CT images and large morphological differences, existing methods generally have the following problems, such as poor detection and segmentation, and the high false-positive rate of lung nodules. In addition, the detection and segmentation of pulmonary nodules are often carried out independently, which leads to an inefficient inspection process.

To tackle these challenging issues, a multiscale fusion method for pulmonary nodule detection is proposed. Aiming at the problem that the detection rate of pulmonary nodules is not high enough, a Faster R-CNN pulmonary nodule detection model which integrates multiscale features is proposed. Combining the feature map which integrates the accurate location information of the bottom layer and the strong semantic information of the high layer to Faster R-CNN, it can provide more effective features for small nodule detection and achieve a higher detection rate. Aiming at the problem that 2D model detection results generally have a higher false-positive rate, a new candidate nodule classification model based on a 3D convolution neural network is proposed. The model contains three branches, which accepts inputs of different scales. Multibranch fusion is carried out to further improve the robustness of spatial context information and then improve the classification accuracy of candidate nodules.

The contributions of this paper are as follows:

1. A new candidate nodule detection model is proposed, which makes full use of Faster R-CNN fused with multiscale features to realize the detection of small nodules on large-scale feature maps. This model effectively improves the detection rate of small-scale nodules
2. A new false-positive nodule filtering model is proposed, which fully utilizes a 3D convolution neural network to encode the multilevel context information of pulmonary nodules to classify the candidate nodules obtained by the candidate nodule detection model, so as to reduce the false-positive nodules and improve the classification performance
3. Experiments are performed with the proposed pulmonary nodule detection model on real datasets, and the experimental results indicate that the proposed method can improve the accuracy of pulmonary nodule detection

## 2. Related Work

### 2.1. Pulmonary Nodule Detection

With the rapid development of deep learning, there are many detection methods of pulmonary nodules based on deep learning in recent two years and have achieved good results. Deng and Chen [13] propose a pulmonary nodule detection algorithm based on deep convolutional neural network, which adds the deep supervision of partial CNN layers innovatively. Sheng [14] develops a computer-aided detection (CADe) scheme using the balanced CNN with classic candidate detection. Masud et al. [15] propose an end-to-end convolutional neural network-based automatic pulmonary nodule detection and classification system which has only four convolutional layers. Chi et al. [16] propose a novel deep convolutional neural network (DCNN) framework for detecting pulmonary nodules in the chest CT image. Tang et al. [17] propose a novel DCNN approach, which uses a U-Net-inspired 3D Faster R-CNN trained using online hard negative mining. Eun et al. [18] propose a novel framework using the ensemble of 2D CNNs using single views, which utilizes single-view 2D patches to improve both computational and memory efficiency. Dodia et al. [19] propose a method to reduce false positive, named receptive field regularized V-net (RFR V-Net). It uses a receptive regularization in the V-Net model, and nodule classification is performed using a new combination of SqueezeNet and ResNet. Zhang et al. [20] propose a 3D feature pyramid network (FPN) for single-stage pulmonary nodule detection, combining the 3D characteristics of computed tomography (CT) image data.

### 2.2. Pulmonary Nodule Segmentation

Tong et al. [21] propose a pulmonary nodule segmentation algorithm based on an improved U-Net, which adds a residual network, and the DSC for nodule segmentation is 73.6%. Dong et al. [22] propose a multiview secondary input residual (MV-SIR) convolutional neural network model for 3D lung nodule segmentation. The MV-SIR model achieves excellent results in the 3D segmentation of pulmonary nodules, with a Dice coefficient of 0.926 and an average surface distance of 0.072. Considering the heterogeneity of pulmonary nodules and the presence of similar visual characteristics between nodules and their surroundings, Wang et al. [23] propose a data-driven model, termed the central focused convolutional neural networks (CF-CNN), to segment pulmonary nodules from heterogeneous CT images. Tang et al. [24] propose an end-to-end DCNN for solving the lung nodule detection and segmentation problem with a CPM score of 87.27% for nodule detection and a DSC score of 83.1% for nodule segmentation on the LIDC-IDRI dataset. Shi et al. [25] propose an automatic segmentation algorithm for lung nodules based on multiscale residual U-Net. The proposed method significantly shortened the recognition time, and the segmentation time is only 3.15 s. Wang et al. [26] propose a multigranularity scale-aware network (MGSA-Net) for accurate pulmonary nodule segmentation, and the Dice similarity coefficient (DSC) is 87.32%, as shown in Table 2.

## 3. Problem Description

For pulmonary nodule detection segmentation, the traditional methods need to segment the lung parenchyma first, which is caused by the insufficient fitting ability of the models. Deep learning has powerful fitting and feature extraction abilities. However, due to the small size of pulmonary nodules and the fact that they occupy only a small part of the lung CT images, the multilayer sampling and pooling operations in convolutional neural networks for feature extraction will lose a lot of nodule location information, making the nodule location difficult to predict. Although more current deep learning models have achieved good results in different target detection tasks, their detection rates are not high for small targets like lung nodules, and a large number of false-positive nodules are included in the detection results. Since pulmonary nodules are similar to many tissues in the lung CT images in terms of shape and gray value, and pulmonary nodules themselves have different morphologies, it is difficult to accomplish the task of false-positive nodule filtering. 2D convolutional neural networks cannot fully utilize the spatial contextual information provided by 3D lung nodules as well as the nodule information between pathological slices. To tackle these challenging issues, Faster R-CNN integrating multiscale features is proposed for candidate nodule detection segmentation, and a 3D convolutional neural network based on multiscale fusion is designed to reduce false-positive nodules.

## 4. Materials and Methods

**Figure 1:**
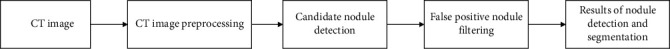
The overall flow diagram.

Figure 1 shows the overall flow diagram of our proposed framework that consists of five parts: CT image, CT image preprocessing, candidate nodule detection, false-positive nodule filtering, and results of nodule detection and segmentation. We describe the CT image preprocessing in Section 4.3.2. In Sections 4.1 and 4.2, we describe the candidate nodule detection and false-positive nodule filtering. Finally, we can get the results of nodule detection and segmentation.

### 4.1. Candidate Nodule Detection Model Based on Multiscale Feature

**Figure 2:**
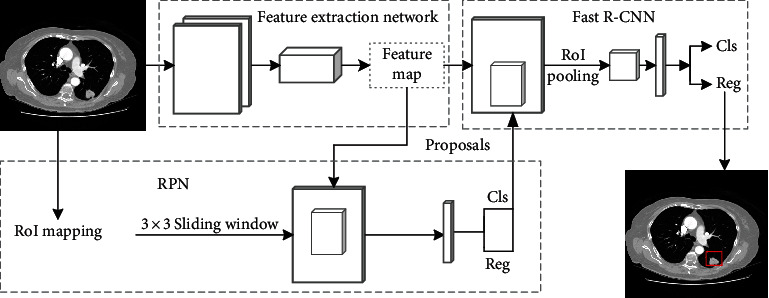
Faster R-CNN nodule detection framework.

The candidate nodule detection framework based on Faster R-CNN is shown in Figure 2. The input of this framework is preprocessed lung CT images, and the output is the probability of the candidate nodule and the position of the bounding box. As can be seen from Figure 2, the detection model mainly includes the following three modules: feature extraction network module, RPN module, and Fast R-CNN module. Firstly, the feature extraction network module extracts features of the input CT image to generate a feature map, which is shared by the RPN module and Fast R-CNN. Then, the RPN module generates some regions of interest. Finally, the regions of interest generated by RPN are input to the Fast R-CNN module for classification and regression. In order to improve the detection rate of small nodules, three modules of the model are improved.

#### 4.1.1. Feature Extraction Network

In order to make the feature map which has more semantic information, ResNet can be used as the feature extraction network, which adds a residual unit to CNN. As shown in equations (1) and (2), the input *x*_*i*_ passes through the mapping network *h*(*x*_*i*_), then combines the output of the feature extraction map *F*(*x*_*i*_, *W*_*i*_) through the residual path in the form of element-wise addition to obtain *y*_*i*_, and obtains the final output *x*_*i*+1_ of the unit through the mapping *f*. The residual link path can effectively solve the problem of gradient disappearance caused by the depth of the network and improve the convergence speed and training efficiency of the network.

(1) $$\begin{array}{c} y_{i}=h\left(x_{i}\right)+F\left(x_{i},W_{i}\right), \end{array}$$

(2) $$\begin{array}{c} x_{i+1}=f\left(y_{i}\right). \end{array}$$

**Figure 3:**
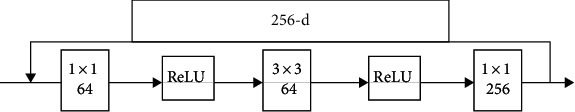
The residual structure.

In order to reduce the amount of calculation, the residual structure shown in Figure 3 is adopted in the implementation of the feature extraction network. Firstly, the input dimension is reduced by 1 × 1 convolution to reduce the number of parameters. Then, a 3 × 3 convolution kernel is used to extract features. Finally, the output dimension is promoted to the original dimension by using 1 × 1 convolution. This implementation is almost the same as the standard residual structure in effect, but the amount of calculation is greatly reduced.

**Figure 4:**
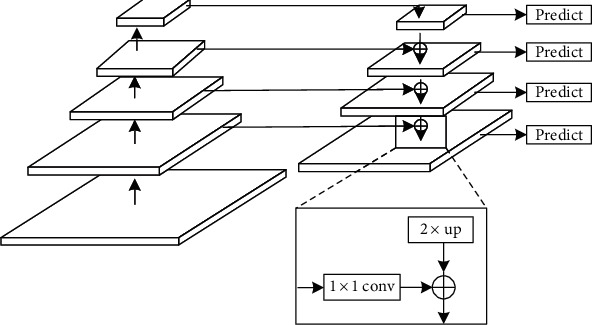
Bottom-up path and top-down path and lateral connection.

In order to improve the detection effect of the detection model based on Faster R-CNN for small nodules, the idea of a feature pyramid is used to generate multiscale feature maps in the part of the feature extraction network, and the overall structure is shown in Figure 4. Feature pyramid network [27] uses the bottom-up path to extract features by the forward propagation, uses the top-down path to upsample the high-level feature map by deconvolution, and realizes lateral connection through 1 × 1 convolution. This network integrates low-level feature maps that have low semantic information and high resolution with high-level feature maps that have high semantic information and bottom resolution, so that the feature map of each scale has rich semantic information.

ResNet-101 is used as the feature extraction network, which includes 5 levels that are recorded as Conv1, Conv2, Conv3, Conv4, and Conv5, and each level outputs feature maps of different scales. The outputs of Conv2, Conv3, and Conv4 are denoted as C2, C3, and C4, respectively, which have convolution steps of 4, 8, and 16 pixels for the input image, respectively. These convolution steps determine the sliding step of the sliding window when the region proposal network generates anchor boxes. Considering the problem of memory consumption, the output of Conv1 is not introduced into the multiscale feature structure, and considering that the maximum size of nodules is only about 40 mm, the output of Conv5 is not introduced into the multiscale feature structure, too. In addition, the number of channels of multiscale features is uniformly set to 256 dimensions; that is, the last 3 × 3 convolutional layers have 256 channels.

The strategy for generating multiscale feature maps is as follows. Firstly, the feature map output by Conv5 is upsampled by deconvolution to obtain a feature map with a twofold size, which is denoted as P4. Secondly, the dimension of C4 is reduced to the same dimension as P4 by 1 × 1 convolution, and then the reduced C4 is superimposed on P4 by adding corresponding elements. Then, P4 is upsampled by deconvolution again to obtain P3, and C3 and P3 are repeatedly connected laterally. What is more, the iteration is repeated until the feature map of the P2 level is generated. Finally, the convolution operation with a convolution kernel size of 3 × 3 is performed on P2, P3, and P4, respectively, to eliminate the aliasing effect caused by the addition of corresponding elements in the horizontal connection. The multiscale feature map can be generated by the above strategy for subsequent RPN module and Faster R-CNN module.

#### 4.1.2. Region Proposal Network

**Figure 5:**
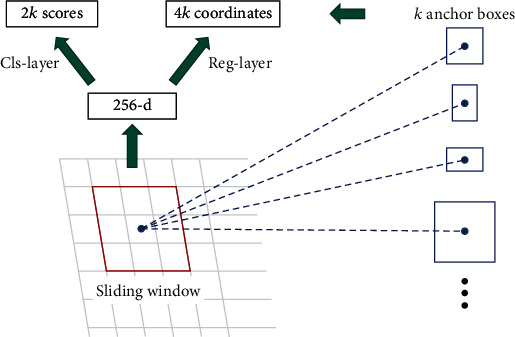
Region proposal network.

Region proposal network (RPN) uses a sliding window to slide on the original image to generate anchor boxes, as shown in Figure 5. The sliding step length is determined by the size mapping relationship between the original image and the feature image. Each sliding generates a series of anchor boxes of different sizes and different aspect ratios in the original image, which are some candidate frames that may contain the target to be detected. The task of the RPN is to filter and adjust these anchor boxes to get the region of interest closer to the target to be detected. Among them, classification and regression are realized by using two 1 × 1 convolutions, so RPN is a fully convolutional network structure, and the network part of classification and regression is called the RPN head network.

The strategy of using multiscale feature maps to replace the single scale feature maps of the original Faster R-CNN is to attach an RPN head network to each scale feature map of multiscale feature maps. Since the head network slides on each position of each scale feature map, it is not necessary to set anchor boxes of different scales on the feature map of a specific scale, only to set anchor boxes of different proportions. Instead of using the traditional method, the *K*-means++ algorithm is used to determine the anchor box, which is shown in Algorithm 1.

The original *K*-means ++ clustering algorithm in Algorithm 1 uses Euclidean distance, but when the size of two anchor boxes is larger, the calculated Euclidean distance is larger than that of small anchor boxes. Therefore, this data cannot accurately reflect the similarity between anchor boxes, and another distance can be used instead of Euclidean distance, which is defined as shown in equation (3):

(3) $$\begin{array}{c} D\left(A,B\right)=1-IoU\left(A,B\right), \end{array}$$

where *IoU*(*A*, *B*) is the intersection over the union of the anchor box *X* and anchor box *Y*, indicating the overlapping degree of two anchor boxes. As shown in equation (4), the larger the intersection over the union ratio is, the greater the overlapping degree of the two anchor boxes is.

(4) $$\begin{array}{c} IoU\left(A,B\right)=\frac{A\cap B}{A\cup B}. \end{array}$$

The most suitable size of the anchor box can be obtained by calculating and rounding by Algorithm 1. In addition, in the forward propagation of the training process, it is necessary to distinguish whether the anchor box belongs to the foreground (target to be detected) or background. The criteria are set as follows: the anchor box with the intersection over the union of the target frame greater than 0.5 is regarded as a positive sample; otherwise, it is regarded as a negative sample. The region of interest closer to the target to be tested is obtained by the above method, which is used for classification and regression by the Fast R-CNN module.

#### 4.1.3. Fast R-CNN Module

The Fast R-CNN module adds the region of interest pooling layer before the fully connected layer, which effectively overcomes the problem of candidate region size variation. Fast R-CNN maps the candidate regions to the feature map, which realizes feature sharing and effectively overcomes the time-consuming problem caused by repeated operations in the previous R-CNN feature extraction process. However, the Fast R-CNN module realizes classification and regression by a fully connected layer, which requires feature maps with the same dimension and the same size, but the bounding box size of the region of interest output by RPN is different. In order to solve this problem, the ROI pooling layer is introduced into the Fast R-CNN module to normalize the size of the ROI.

**Figure 6:**
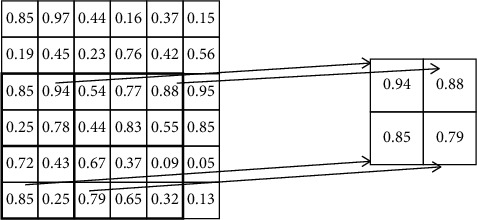
The pooling of ROI.

The pooling of ROI is shown in Figure 6. Firstly, ROI pooling maps the region of interest from the original image to the corresponding position of the feature map according to the mapping relationship. Then, the mapped region is divided into subregions according to the preset size. Finally, the maximum pooling operation is performed for each subregion.

In order to normalize features of ROI on the multiscale feature map, it is necessary to assign the region of interest of different scales to feature maps of different scales. The region of interest with the width of *w* and height of *h* (relative to the scale of the input image) can be assigned to feature maps of *P*_*k*_ scale by formula (5).

(5) $$\begin{array}{c} k=\left\lfloor k_{0}+\log_{2}\left(\frac{\sqrt{wh}}{40}\right)\right\rfloor . \end{array}$$

*k*_0_ is set to 4. If the scale of ROI becomes smaller, such as 1/2 of 40, then *k* = 4 + log_2_ (1/2) = 3; that is, it should be mapped to a more refined level P3.

Feature vectors composed of all candidate regions obtained from the pooling layer of ROI are sent to the fully connected layer and softmax layer to calculate the category of each candidate frame and output the category score of them. At the same time, the offset prediction value of each candidate region relative to the actual position is obtained by using the regression algorithm again, and the candidate frame is modified to obtain a more accurate target frame and complete the detection of candidate pulmonary nodules.

### 4.2. False-Positive Filtering Model Based on Multiscale Fusion

2D convolution neural network ignores the relevant information between slices because of the three-dimensional structures of pulmonary nodules and cannot make full use of the spatial context information. 3D convolutional neural network not only extracts high-level abstract features but also makes full use of spatial context information to make up for the above shortcomings. Therefore, a multiscale merge convolutional neural network (MSM-CNN) algorithm based on a 3D convolutional neural network is proposed to filter false-positive pulmonary nodules.

#### 4.2.1. Network Structure

**Figure 7:**
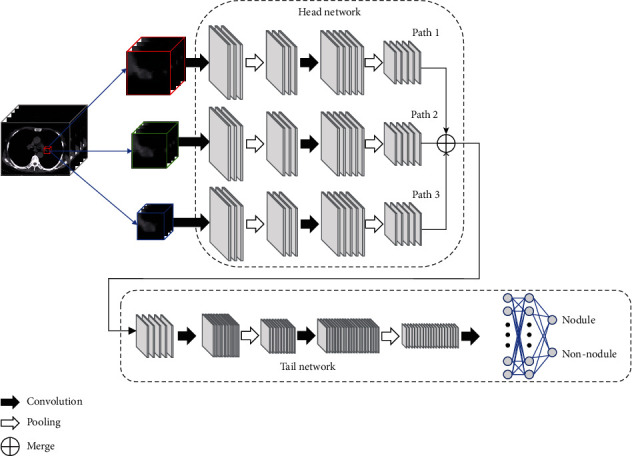
The MSM-CNN.

In the network layer operation, multichannel feature maps are generated to encode different features. Different from 2D CNN, the feature map of 3D CNN is a three-dimensional feature body, rather than the two-dimensional planar feature map of 2D CNN. The false-positive filtering model MSM-CNN is built by 3D CNN, and its structure is shown in Figure 7. The model is divided into two parts. The first part is a three-path network with three branches, which is called the head network, and the second part is the shared network part after feature fusion, which is called the tail network.

In the head network, each path uses 3D patch cubes of different size nodules as input, and the three branch networks Path1, Path2, and Path3 are all composed of two 3D convolutional layers and two 3D pooling layers. In the tail network, two 3D convolution layers and two 3D pooling layers are shared by the feature map generated after the fusion of the three-branch network. Each convolution layer and pooling layer are regarded as a group, followed by the ReLU activation function layer. Then, they are activated by two fully connected layers and two softmax function activation layers of neurons. The detailed parameters are shown in Table 3.

The design inspiration of MSM-CNN network layer is from VGG-Net [28], and its specific structure mainly includes the following parts. Firstly, in order to reduce the number of parameters of the model, a 3 × 3 × 3 3D convolution kernel is used as a feature extractor in the 3D convolution layer. The 3D convolution kernel sweeps through the input cubes of the previous layer in step 1 to get the characteristics of different channels, then adds a bias term, and uses the nonlinear activation function to improve the fitting ability of the network. Then, each 3D convolutional layer is followed by a 3D maximum pooling layer, which is used to downsample the 3D feature block to obtain local translation invariance in 3D space and further reduce the number of parameters, the number of calculations, and the risk of overfitting. Finally, in the fully connected layer, different from the local connection in the convolutional layer, the neuronal connection is denser than the connection in the convolutional layer.

Specifically, each neural node in the fully connected layer is connected to all neural nodes in the adjacent layer, and these dense connections can make the extracted features have stronger expression ability. In order to realize the fully connected layer, the feature block is expanded to the one-dimensional vector at first; secondly, the vector matrix multiplication is performed, and then, a deviation term is added to it, and finally, the nonlinear function ReLU is used to activate it.

#### 4.2.2. Fusion Strategy

The fusion strategy includes feature fusion and model fusion, which are fusion methods at different levels. At the level of feature fusion, the MSM-CNN model uses the method of adding feature map elements point by point in the merged layer for feature fusion. The fusion process is defined as shown in formula (6):

(6) $$\begin{array}{c} F_{\text{add}}=\overset{3}{\underset{k=1}{\sum }}W_{k}. \end{array}$$

*k* is the number of branches, *W*_*k*_ is the parameter of the feature block, and *F*_add_ is the fused feature. Since these feature blocks are all extracted from different size blocks of the same nodule, the output semantic information is the same, and they are all constrained by the loss of the same label, so this method is used to fuse features to achieve the effect of enhancing semantic information.

**Figure 8:**
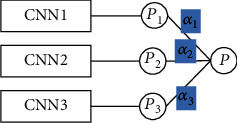
The schematic diagram of model fusion.

At the level of model fusion, the three branches of the head network of MSM-CNN, Path1, Path2, and Path3, can form separate networks with the tail network, which are, respectively, denoted as CNN1, CNN2, and CNN3. In order to combine the prediction results of CNN1, CNN2, and CNN3, the mechanism of the AdaBoost weighted voting fusion model is used here for reference, as shown in Figure 8. The idea of it, which is called Fusion-CNN, is to assign a smaller weight to the model with a poor classification effect and assign a larger weight to the model with a good classification effect. Weight *α* is, respectively, assigned to CNN1, CNN2, and CNN3, and its calculation formula is as equation (7):

(7) $$\begin{array}{c} \alpha =\frac{1}{2}\ln\left(\frac{1-\varepsilon }{\varepsilon }\right). \end{array}$$

*ε* is the misclassification rate of the model, that is, the proportion of the number of samples with classification errors in the total number of all test samples.

In the experiment, the strategy for determining *α* is as follows: during training, the strategy of crossvalidation is adopted, and a set of weights *α*_1_, *α*_2_, and *α*_3_ can be obtained after each round of the experiment. All the weight values in the last round of crossvalidation are saved, their mean values are calculated, respectively, and finally, the weights *α*_1_, *α*_2_, and *α*_3_ of each model are determined. During testing, input candidate nodule data for testing, and each model outputs the corresponding prediction probabilities p_1_, p_2_, and p_3_ and then uses the weights saved during training as the weights of each model. The final prediction result is obtained by the weighted sum of the output probability of the three models; that is, the final prediction probability is *p* = *α*_1_ × *p*_1_ + *α*_2_ × *p*_2_ + *α*_3_ × *p*_3_.

### 4.3. Materials and Data Processing

#### 4.3.1. Data Materials

LUNA16 (LUng Nodule Analysis 2016) public dataset is used as experimental data. The data of LUNA16 are derived from a larger public dataset LIDC-IDRI (https://wiki.cancerimagingarchive.net/display/Public/LIDC-IDRI), which contains 1018 CT scans, that is, 1018 cases. In the LUNA16 dataset, CT samples with slice thickness greater than 3 mm in LIDC-IDRI dataset are removed, CT samples with inconsistent slice pixel intervals and missing portions of the slice are also removed, and finally, 888 CT samples are retained. A total of 1186 nodules are labeled in the LUNA16 dataset with nodule diameters ranging from 3 mm to 40 mm. Positive nodule and negative nodule samples each account for approximately one-half, and 2795 nodules are obtained after data augmentation.

#### 4.3.2. CT Image Preprocessing

Firstly, in order to make full use of the information of the adjacent three slices, the slices superimposed on the center of the nodule, and the two adjacent slices constitute a three-channel image, which can make use of the pretraining parameters in the detection network, so as to reduce the training difficulty and training time. Secondly, in order to reduce the parameter search space of the candidate nodule detection model, the pixel values of CT images are intercepted with a [-1200, 600] range window to preserve complete lung tissue, and the *Z*-score algorithm is used to normalize it.

Then, as the pulmonary nodule detection system requires that the position of the pulmonary nodule in the input is marked based on the volume pixel coordinate system, while the position information of pulmonary nodules in the original data are marked based on the world coordinate system, so the coordinate system is transformed. As the CT images collected by different instruments in different environments have great differences in image pixel spacing when processing the CT images which have transformed coordinate system, the three dimensions should be interpolated to 1 mm × 1 mm × 1 mm, and the size should be scaled to 512 × 512.

Finally, two stages of data augmentation are carried out. In the stage of candidate nodule detection, for nodules with a diameter greater than 20 mm, four adjacent sections (two upper and two lower) are taken as new data to describe the same pulmonary nodule. For nodules with a diameter of more than 10 mm and less than 20 mm, two adjacent sections (one upper and one lower) are taken in the same way for augmentation. For nodules with a diameter of less than 10 mm, no augmentation is made. In the stage of false-positive nodule filtering, 70 × 70 × 70, 36 × 36 × 36, and 20 × 20 × 20 nodules are cut out for each candidate nodule based on the nodule center, and then nodules of these three scales are randomly cut out into 64 × 64 × 64, 32 × 32 × 32, and 16 × 16 × 16. Since not all the detected candidate nodules take the center of the nodule as the image center, this method can enrich the position distribution of nodules in nodule images and increase the number by 25 times for each candidate nodule. Then, each candidate nodule is turned from *X*, *Y*, and *Z* three axes, and the number of samples can be expanded by another 8 times. Finally, 200 times of nodules are obtained. After the above two steps of augmentation, the quantity ratio of positive samples (true nodules) and negative samples (nonnodules) is about 2 : 5, and double repeated sampling is used for true positive nodules during training to balance the number of positive samples and negative samples in the training set.

## 5. Results and Discussion

### 5.1. Experiment Details

The experimental platform is Google Colab, the server system is Ubuntu, and the GPU is K80 (video memory 12 GB). The network parameters are randomly initialized with a Gaussian distribution with a mean value of 0 and a standard deviation of 0.01. The stochastic gradient descent method with momentum is used to update the model parameters. The random gradient descent with momentum (SGDM) is used to update model parameters, the momentum coefficient is set to 0.9, and the batch size is set to 64. The weight attenuation coefficient is set to 0.0005, which can alleviate the phenomenon of overfitting. Using the dropout strategy, the dropout rate is set to 0.5, which can also alleviate overfitting.

The most suitable sizes of anchor boxes in RPN are calculated and rounded by Algorithm 1, which are set to 4 × 5, 6 × 8, 10 × 8, 12 × 16, 18 × 15, 17 × 20, 20 × 26, 26 × 30, and 36 × 30, respectively. The gradient descent method is used to update the parameters of the neural network, and the most critical hyperparameter in the gradient descent method is the learning rate, which is initially set to 0.001, and then gradually reduced after 20000 steps of iteration. The MSM-CNN model has inputs of three scales. According to the statistical information, the three scales of a 3D patch of nodules can be determined, which are set to 16 × 16 × 16, 32 × 32 × 32, and 64 × 64 × 64, respectively.  Table 4 shows the experiment details.

### 5.2. Results

#### 5.2.1. Candidate Nodule Detection

**Figure 9:**
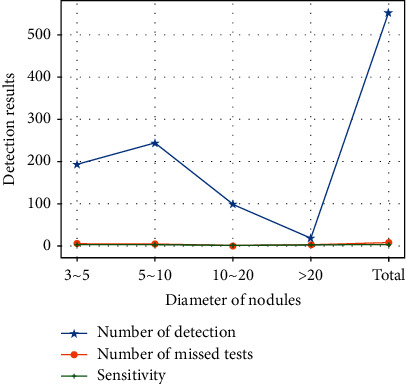
Detection results of multiscale Faster R-CNN.

Table 5 shows the detection results of the pulmonary nodule detection model based on Faster R-CNN. The detection network of this model is a single-scale feature detection network based on ResNet.  Table 6 and Figure 9 show the detection results of the pulmonary nodule detection model based on Faster R-CNN integrating multiscale features. Both tables are the results of the same average number of candidate nodules, so they have the objectivity of contrast sensitivity.

It can be seen from these two tables that both methods have a high detection rate for large-scale pulmonary nodules (more than 10 mm). The advantage of the Faster R-CNN integrating multiscale features is reflected in the detection of small nodules, especially for nodules of 3~5 mm, and the proposed model has increased the sensitivity by more than 10% compared with the other single-scale model, which also verifies the importance of multiscale features for small nodules detection, and achieves the goal of improving the detection rate of small-scale pulmonary nodules. All of this can prove that the sensitivity of the Faster R-CNN model with multiscale features for small nodules is better than that of the Faster R-CNN model with single-scale features.

#### 5.2.2. False-Positive Nodule Filtering

**Figure 10:**
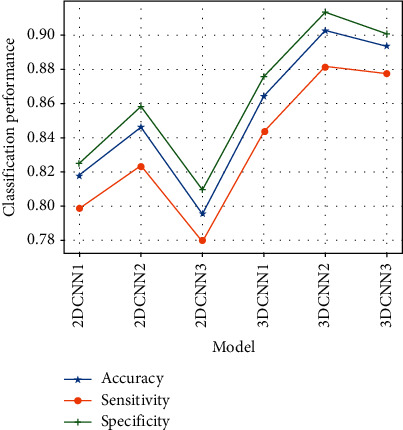
The classification performance comparison of single branch models.

First, the classification performance of single branch models is compared. In this experiment, three branches of the MSM-CNN head network, Path1, Path2, and Path3, are combined with the tail network to form separate networks, which are, respectively, recorded as CNN1, CNN2, and CNN3. Then, the 2D convolutional neural network and 3D convolutional neural network are, respectively, used to implement these three subnetwork models, which are used as separate classifiers to evaluate their classification performance, as shown in Table 7 and Figure 10.

The accuracies of single branch models 2D CNN1, 2D CNN2, and 2D CNN3 (input sizes of 16 × 16, 32 × 32, and 64 × 64, respectively) obtained by using 2D convolutional neural networks are 81.76%, 84.67%, and 79.54%, respectively, and the accuracies of single branch models 3D CNN1, 3D CNN2, and 3D CNN3 (input sizes of 16^3^, 32^3^, and 64^3^, respectively) obtained by using the 3D convolutional neural network are 89.35%, 90.24%, and 86.43%, respectively.

It is clear that the classification effect of 3D CNN is better than that of 2D CNN as a whole. This is because 3D CNN can make better use of the spatial context information of nodules and extract more discriminative features, which also proves the advantages of the 3D convolutional layer. For the network in the same dimension, the classification effect of the network with medium-scale input is better than that of the other two networks, because larger scale images contain complete nodules, but introduce more background interference information to affect the classification effect. The smaller scale images cannot contain the global information for larger nodules, so the classification effect for large-scale nodules is general. The medium-scale images can cover the global information of nodules of all sizes and provide rich background information for small nodules, so the classification effect is better.

**Figure 11:**
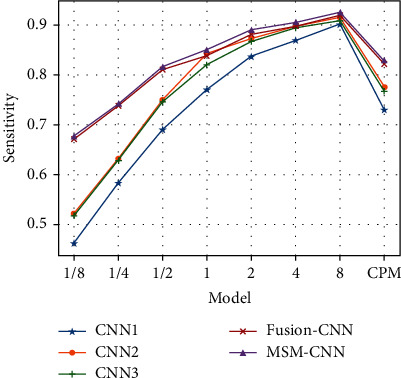
Sensitivity and competition performance metric of different 3D models.

Next, the classification performance of fusion branch models is compared, and the sensitivity and competitive performance metric of different 3D models is compared too. The results are shown in Table 8 and Figure 11.

**Figure 12:**
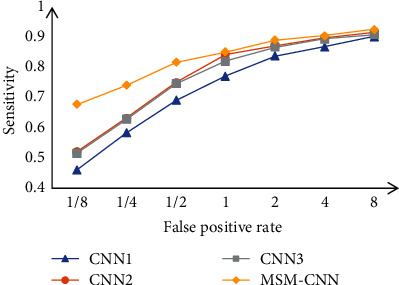
FROC curves of different models.

It can be clearly seen that the CPM values of two models with different fusion methods are higher than that of the single branch models, indicating that the multilevel context features can be learned by combining the three branches, thus improving the classification accuracy and ensuring both low false-positive and high sensitivity. In addition, the CPM value of the merge method is higher than the fusion method, which shows that the merge method can extract more robust features and improve the classification effect from the feature level compared with the simple model fusion.  Figure 12 is a contrasting figure of FROC curves of different 3D models in Table 8.

From Figure 12, the excellent performance of the MSM-CNN model can be seen more clearly, and under more stringent threshold requirements, the effect of MSM-CNN is much better than that of the single model.

Finally, the performance comparison experiment of the whole model is carried out, which compares the performance of different false-positive filtering methods, and the results are shown in Table 9. SVM classifiers solve the problem of pulmonary nodule detection by cascading and achieving a sensitivity of 85.9% at the level of 2.5 false-positive nodules per scan. Torres et al. [29] combine two subsystems based on segmentation and voxel, using a feedforward neural network for classification by 13 features, and achieve a sensitivity of 80% at the level of 8 false-positive nodules per scan. Brown et al. [30] propose a new automated pulmonary nodule detection system based on intensity threshold, Euclidean distance transformation, and watershed-based segmentation, which achieves a sensitivity of 75% at the level of 4 false-positive nodules per scan. Extract the coronal planes, sagittal planes, and axial planes of each candidate nodule detected by the candidate nodule detection module of the OverFeat model and finally achieve a sensitivity of 76% at the level of 4 false-positive nodules per scan. Setio et al. [31] extract 9 sections of candidate nodules, use these section data to train multiple 2D CNN networks, and finally achieve a sensitivity of 90.1% at the level of 4 false-positive nodules per scan.

The proposed method finally achieves a sensitivity of 90.5% at the level of 4 false-positive nodules per scan, and the CPM score is 0.829. Both results are higher than methods in other works of literature, which verifies the effectiveness of the proposed 3D CNN framework with multiscale fusion, MSM-CNN, in the false-positive filtering stage.

## 6. Conclusions

A multiscale fusion method for pulmonary nodule detection is proposed. In the stage of candidate nodule detection, some improvements have been made based on the mainstream universal detection framework Faster R-CNN that multiscale feature maps are used in the feature extraction network, and the *K*-means ++ clustering method is recommended to determine the scale and proportion of the anchor box in the module of regional proposal network. In the stage of false-positive nodule filtering, a multiscale false-positive filtering model based on a 3D convolutional neural network, MSM-CNN, is proposed, which can simultaneously receive 3D slices of the same nodule at different scales as input. We conduct comparative experiments with some traditional methods, and the experimental results show that the proposed method has greater improvement in the overall detection rate of pulmonary nodules, especially in the detection rate of small nodules, and further improves the accuracy of the classification of true and false-positive pulmonary nodules.

## 7. Future Work

Our proposed method only performed detection and segmentation, lacking diagnosis of benign and malignant. However, the diagnosis of benign and malignant plays an important role in the treatment of lung cancer. If pulmonary nodules can be accurately classified as benign or malignant at the same time of detection and segmentation, it can further help physicians in the diagnosis of lung cancer and reduce their workload. Therefore, in future research work, we should try to detect, segment, and diagnose benign and malignant simultaneously. In addition, the existing methods only stay at the semantic segmentation stage and do not achieve instance segmentation. Therefore, the instance segmentation of candidate nodes is also a future research direction.

## Figures and Tables

**Algorithm 1:**
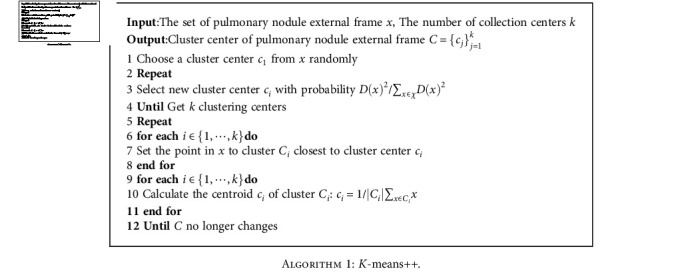
*K*-means++.

**Table 1:** Abbreviation table.

Abbreviation	Full name
CT	Computer tomography
CNN	Convolutional neural network
RPN	Region proposal network
Faster R-CNN	Faster region convolutional neural network
DCNN	Deep convolutional neural network
DSC	Dice similarity coefficient
CPM	Competition performance metric
MSM-CNN	Multiscale merge convolutional neural network

**Table 2:** Related work.

Authors	Methods	Field
Deng and Chen [13]	CNN	Pulmonary nodule detection
Sheng [14]	CNN	Pulmonary nodule detection
Masud et al. [15]	CNN	Pulmonary nodule detection
Chi et al. [16]	CNN	Pulmonary nodule detection
Tang et al. [17]	U-Net-inspired 3D Faster R-CNN	Pulmonary nodule detection
Eun et al. [18]	2D CNN	Pulmonary nodule detection
Dodia et al. [19]	RFR V-Net	Pulmonary nodule detection
Zhang et al. [20]	3D FPN	Pulmonary nodule detection
Tong et al. [21]	U-Net	Pulmonary nodule segmentation
Dong et al. [22]	3D MV-SIR	Pulmonary nodule segmentation
Wang et al. [23]	CF-CNN	Pulmonary nodule segmentation
Tang et al. [24]	CNN	Pulmonary nodule segmentation
Shi et al. [25]	U-Net	Pulmonary nodule segmentation
Wang et al. [26]	MGSA-Net	Pulmonary nodule segmentation

**Table 3:** Network structure of the three-dimensional candidate nodule filtering model.

Layer	Path1		Path2		Path3	
	Kernel	Output	Kernel	Output	Kernel	Output
Input	—	16^3^, 1	—	32^3^, 1	—	64^3^, 1
C1	3^3^, 1	16^3^, 32	3^3^, 1	32^3^, 32	3^3^, 1	64^3^, 32
M1	1^3^, 1	16^3^, 32	2^3^, 2	16^3^, 32	2^3^, 2	32^3^, 32
C2	3^3^, 1	16^3^, 64	3^3^, 1	16^3^, 64	3^3^, 1	32^3^, 64
M2	1^3^, 1	16^3^, 64	1^3^, 1	16^3^, 64	2^3^, 2	16^3^, 64
Merge	Output: 16^3^, 64					
C3	Kernel: 3^3^, 1; output: 16^3^, 128					
M3	Kernel: 2^3^, 2; output: 8^3^, 128					
C4	Kernel: 3^3^, 1; output: 8^3^, 256					
M4	Kernel: 2^3^, 2; output: 4^3^, 256					
FC1	1024					
FC2	2					
Soft	2					

**Table 4:** Experiment details.

Experimental items	Parameter
Platform	Colab
Server system	Ubuntu
GPU	K80
Momentum coefficient	0.9
Batch size	64
Weight attenuation coefficient	0.0005
Dropout rate	0.5
Anchor box sizes	4 × 5, 6 × 8, 10 × 8, 12 × 16, 18 × 15, 17 × 20, 20 × 26, 26 × 30, 36 × 30
Learning rate	0.001
Scales of 3D patch of nodules	16 × 16 × 16, 32 × 32 × 32, 64 × 64 × 64

**Table 5:** Detection results of single-scale Faster R-CNN.

Diameter of nodules (mm)	Number of detections	Number of missed tests	Sensitivity
3~5	168	30	84.8%
5~10	236	10	95.9%
10~20	96	1	99.0%
>20	18	0	100%
Total	518	41	92.7%

**Table 6:** Detection results of multiscale Faster R-CNN.

Diameter of nodules (mm)	Number of detections	Number of missed tests	Sensitivity
3~5	193	5	97.4%
5~10	243	3	98.8%
10~20	97	0	100%
>20	18	0	100%
Total	551	8	98.6%

**Table 7:** The classification performance comparison of single branch models.

Model	Accuracy	Sensitivity	Specificity
2D CNN1 (16 × 16)	81.76%	79.87%	82.45%
2D CNN2 (32 × 32)	84.67%	82.34%	85.77%
2D CNN3 (64 × 64)	79.54%	77.94%	81.03%
3D CNN1 (16^3^)	86.43%	84.33%	87.56%
3D CNN2 (32^3^)	90.24%	88.13%	91.31%
3D CNN3 (64^3^)	89.35%	87.72%	90.08%

**Table 8:** Sensitivity and competition performance metric of different 3D models.

Model	CNN1	CNN2	CNN3	Fusion-CNN	MSM-CNN
1/8	0.461	0.521	0.516	0.671	0.677
1/4	0.583	0.632	0.627	0.737	0.741
1/2	0.69	0.75	0.745	0.81	0.816
1	0.77	0.843	0.82	0.838	0.85
2	0.837	0.872	0.867	0.881	0.89
4	0.868	0.898	0.894	0.897	0.905
8	0.902	0.916	0.909	0.92	0.925
CPM	0.73	0.776	0.768	0.822	0.829

**Table 9:** The classification performance comparison with other methods.

Method	Sensitivity (FP/scan)	CPM
Torres et al. [29]	80.0/8	0.742
Brown et al. [30]	75.0/4.0	0.748
Setio et al. [31]	90.1/4.0	0.814
Mei et al. [32]	89.5/4.0	0.822
Dodia et al. [19]	89.2/4.0	0.816
Fusion-CNN	89.7/4.0	0.822
MSM-CNN	90.5/4.0	0.829

## Data Availability

The data used to support the findings of this study are included within the article.
